# The Convention of Medical Colleges

**Published:** 1876-06

**Authors:** 


THE CONVENTION OF MEDICAL COLLEGES.—So much has been said, for years past, of reform in medical education, and so little accomplished, that the profession seems, to some extent, to have lost hope of resulting good. For our part we have never tired of the cry. All revolutions, or grand changes, in any important department of the world's advance, can be accomplished only by years of thought and effort, and volumes of lines that seem to fall unheeded by the wayside. It requires such time and effort to ripen and mature opinion requisite to fulfil its object. Hence we never see an address, report, essay, on the subject, stale and worn though the subject seems, but we feel that it is one of the elements for ripening sentiment, and preparing the professional mind for the needed change.

It is generally known that a convention of medical colleges has been called to assemble at Philadelphia a few days in advance of the meeting of the American Medical Association. It is impossible to say what the result of that convention may be. It is not by any means the first effort of a similar character. Previous ones have failed of practical good, as may this one. Even should there be such failure, we will not be discouraged. The effort itself, with the discussion and agitation of the principle involved, must prove a long stride toward the good that must be some day reached.

But we are very hopeful of some decided reform emanating from this convention, sooner or later. The profession is ready for it. The schools desire it. The country demands it. This change of the system of teaching must be wrought within and by the profession. We cannot look for it to the firm, single, and overruling hand of government, as is the case in some of the countries of the old world. The nature of our civil law forbids this.

American medicine and surgery, in its highest type, we firmly believe, is not one whit behind that of the old world. Indeed, in some respects, it surpasses it. But the desire is, to make that type more nearly uniform; or, in plain words, to have fewer inferior doctors; to require all to be more advanced toward that higher type before they can claim a place in the profession.

The call for delegates to this congress requested the schools to clothe the delegates with power to *act*. This request, we hope, will be complied with. It will be perceived that more time has been allowed for the sitting of the convention than has been heretofore available, in order that the matter may be maturely considered in all its bearings.

There are two or three ideas that naturally present themselves in connection with the subject. The one that has been most often urged, is to separate the teaching and the diploma-granting power. At the same time, the difficulty of securing this end is very great. That power of granting diplomas must be cautiously placed. It cannot be placed in the hand of state or national governments. Medical education cannot thrive when subjected to the changes, wrangling, and corruption of politics. Have we ever known a state government to undertake the regulation of medical matters, but that the result was a ridiculous farce, or a more serious calamity? Legislators and governors have no conception of what constitutes a physician. A board of examiners, appointed and controlled by such authority might possibly be composed of a wire-pulling, button-holing, set of inferior men, rather than more worthy and modest men of science. Of course we do not say that such would always be the case. We only say that there could be no security against this possibly contingency.

Nor could this power of controlling diplomas be vested in State or National Medical Associations. It would be but a prolific source of rings and cliques, and add to the difficulty, already great enough, of preserving peace and harmony in these organizations.

Finally, the most available deposit for this power we believe is in the colleges themselves—not in individual colleges, as now, for it is this privilege, or its abuse we would suppress—but in a general Association of Colleges. There is no lack of desire on the part of teachers to make the needed changes. It is the lack of power. No individual school could undertake it, though it would cordially and heartily welcome the plan that would apply to all.

We do not offer this as a perfect plan, or one free from dangers and abuses, but only as one involving the principle that we believe must control the matter.

Other objectionable details in the present plan of teaching medicine it is unnecessary to discuss. They are such as can probably be more easily rectified by the convention than the greater one already alluded to.

We do hope it may be possible to prevent incompetent men entering the *student's* ranks, as well as the ranks of the profession. This matter is at present controlled by the preceptors, and they certainly need reform as much as do the colleges. It is better to stop an incompetent student at the very outset, than to trust to any "preliminary examination" to do so, after he has already expended time and money.

Very likely there will be a change made by this congress in tuition fees. There is nothing gained, to any one, by "beneficiaries," or by ridiculously low fees. They are but too often an invitation for those to become students who would succeed better in pursuits requiring less learning; and those who have the necessary capacity, but for whose entrance these low fees are requisite, would better seek a field more lucrative than the medical profession.

Since writing the above, we learn that the Convention of Colleges met in Philadelphia on the 2d of June, and that twenty-five or thirty colleges were represented, either by letters or delegates. We further learn that quite a number of subjects in which both the profession and the colleges are more or less interested were brought before the convention for discussion and action, but that few, however, were acted on; the majority having been deferred, and will come up for action at the next meeting of the convention.

We are pleased to learn that the necessary steps were taken to make it a permanent organization. This we regard as of the greatest importance, as but little good could be expected from such an organization unless it is permanent, with annual sessions.

The wholesale distribution of beneficiary scholarships, as practiced by some of the schools, was thoroughly discussed and unanimously condemned by the convention.

We regret to learn that the convention failed to pass a resolution that was presented for action, recommending that the fees in medical colleges be more uniform, and that the minimum fee for a course of lectures be one hundred dollars; but we are pleased to know that the course pursued by the faculty of the University of Michigan, in regard to the homœopathic feature recently incorporated in that institution was unanimously condemned.

We learn that the meeting was harmonious, and that much good is expected from its deliberations.

The next meeting of the convention will be held in Chicago, a few days preceding the meeting of the American Medical Association, in June next.

